# Protocol for a multi-centre pilot and feasibility randomised controlled trial with a nested qualitative study: rehabilitation following rotator cuff repair (the RaCeR study)

**DOI:** 10.1186/s13063-019-3407-3

**Published:** 2019-06-06

**Authors:** Chris Littlewood, Marcus Bateman, Kendra Cooke, Susie Hennnings, Tina Cookson, Kieran Bromley, Martyn Lewis, Lennard Funk, Jean Denton, Maria Moffatt, Rachel Winstanley, Saurabh Mehta, Gareth Stephens, Lisa Dikomitis, Linda Chesterton, Nadine E. Foster

**Affiliations:** 1 Arthritis Research UK Primary Care Centre, Research Institute for Primary Care and Health Sciences and Keele Clinical Trials Unit, Keele University, Staffordshire, UK; 2 Derby Shoulder Unit, University Hospitals Derby & Burton NHS Foundation Trust, Derby, UK; 3 Sandbach, UK; 4 Wrightington, Wigan and Leigh NHS Foundation Trust, Wigan, UK; 5 The Robert Jones and Agnes Hunt Orthopaedic Hospital NHS Foundation Trust, Oswestry, UK; 6 Royal Stoke University Hospital, University Hospitals of North Midlands NHS Trust, Stoke, UK; 7 The Royal Orthopaedic Hospital NHS Foundation Trust, Birmingham, UK; 8 Arthritis Research UK Primary Care Centre, Research Institute for Primary Care and Health Sciences and School of Medicine, Keele University, Staffordshire, UK

**Keywords:** Rehabilitation, Physiotherapy, Exercise, Rotator cuff, Shoulder, Randomised controlled trial

## Abstract

**Background:**

Shoulder pain is a highly prevalent complaint and disorders of the rotator cuff, including tears, are thought to be the most common cause. The number of operations repair the torn rotator cuff has risen significantly in recent years. While surgical techniques have progressed, becoming less invasive and more secure, rehabilitation programmes have remained largely like those initially developed when surgical techniques were less advanced and more invasive. Uncertainty remains in relation to the length of post-surgical immobilisation and the amount of early load permitted at the repair site. In the context of this uncertainty, current practice is to follow a generally cautious approach, including long periods of immobilisation in a sling and avoidance of early active rehabilitation. Systematic review evidence suggests early mobilisation might be beneficial but further high-quality studies are required to evaluate this.

**Methods/design:**

RaCeR is a two-arm, multi-centre pilot and feasibility randomised controlled trial with nested qualitative interviews. A total of 76 patients with non-traumatic rotator cuff tears who are scheduled to have a surgical repair will be recruited from up to five UK NHS hospitals and randomly allocated to either early patient-directed rehabilitation or standard rehabilitation that incorporates sling immobilisation. RaCeR will assess the feasibility of a future, substantive, multi-centre randomised controlled trial to test the hypothesis that, compared to standard rehabilitation incorporating sling immobilisation, early patient-directed rehabilitation is both more clinically effective and more cost-effective. In addition, a sample of patients and clinicians will be interviewed to understand the acceptability of the interventions and the barriers and enablers to adherence to the interventions.

**Discussion:**

Research to date suggests that there is the possibility of reducing the patient burden associated with post-operative immobilisation following surgery to repair the torn rotator cuff and improve clinical outcomes. There is a clear need for a high-quality, adequately powered, randomised trial to better inform clinical practice. Prior to a large-scale trial, we first need to undertake a pilot and feasibility trial to address current uncertainties about recruitment, retention and barriers to adherence to the interventions, particularly in relation to whether patients will be willing to begin moving their arm early after their operation.

**Trial registration:**

ISRCTN Registry, 18357968. Registered on 10 August 2018.

**Electronic supplementary material:**

The online version of this article (10.1186/s13063-019-3407-3) contains supplementary material, which is available to authorized users.

## Background

Shoulder pain is a highly prevalent complaint and disorders of the rotator cuff (RC) are thought to be the most common cause [1]. Overall, the prevalence of RC abnormalities, including RC tears, increases with age, from 9.7% in patients under 20 years to 62% in patients over 80 years [2]. Typically, shoulder pain is initially treated using non-surgical means, including, for example, pain medication and physiotherapist-led exercise. However, if symptoms persist and the patient has a RC tear confirmed on a scan, then surgery might be considered [3]. In 2017/18, 18,237 patients with RC syndrome, including tears, were admitted to hospital in the UK’s National Health Service (NHS) [4].

Surgical techniques to repair the RC have progressed over time. They have become less invasive and the repairs have become more secure, raising the possibility of more rapid patient recovery [5, 6]. Despite surgical progression, our understanding of the optimal approach to post-operative rehabilitation, a critical component of the recovery process, is poor [6]. Rehabilitation programmes have remained largely like those initially developed when surgical techniques were less advanced and more invasive [7]. There is uncertainty about two related issues: (1) the length of post-surgical immobilisation and (2) the amount of early load permitted at the repair site [3]. In the context of this uncertainty, current practice is to follow a generally cautious approach, including long periods of immobilisation in a sling (of more than 1 month) and avoidance of active rehabilitation or movement [7], possibly due to fear of failure or re-tear of the repair site. However, such long periods of shoulder immobilisation impact significantly on patient’s quality of life, including their ability to care for themselves, drive and work.

Furthermore, long periods of immobilisation of the shoulder after surgery may result in stiffness and weakness and delay functional recovery, and, moreover, they may actually be detrimental to healing. Improved clinical outcomes, including reduced length of hospital stay, have been reported in other areas of orthopaedic and musculoskeletal rehabilitation with early mobilisation, for example for ankle sprains and joint replacements, without compromising healing [8, 9]. A systematic review and meta-analysis of 12 studies concluded that concerns about the early initiation of rehabilitation following RC surgical repair might not be warranted in terms of adverse patient-reported outcomes or tendon re-tear, but these trials were heterogeneous and too small to detect small to moderate differences between groups [10]. Seven of the 12 studies [9, 11–16] evaluated early versus delayed initiation of rehabilitation. Typically, this referred to initiation of passive range of movement exercise (i.e. with no or minimal load), with the exception of Klintberg et al. [9], who commenced low-level active range of movement exercises from day 2 post-operatively. These studies suggest that early initiation of rehabilitation does not adversely affect pain and disability outcomes in the short (3 months), mid (6 months) or long term (12 months). However, one randomised controlled trial (RCT), regarded as low quality due to lack of both allocation concealment and intention-to-treat analysis, suggested that early initiation of rehabilitation might favourably affect pain and disability outcomes in the short term (4 months) [13]. Five of the 12 studies [11, 12, 14–16] (*n* = 469) evaluated early versus delayed initiation of rehabilitation and reported outcomes in terms of rate of RC tendon re-tear. The pooled odds ratio of tendon re-tear in the early rehabilitation group was 1.3 (95% confidence interval: 0.72–2.2; *p* = 0.41), which suggests that patients undergoing early rehabilitation were slightly more likely to have a tendon re-tear than those in the delayed rehabilitation group but the difference was not statistically significant. Subsequent to the systematic review, a Canadian RCT (*n* = 189) compared early rehabilitation (patient-led mobilisation or movement of the shoulder) with sling immobilisation following RC repair [17]. This trial did not restrict inclusion by size of RC tear and concluded that the range of movement of the shoulder was better at 6 weeks in the early rehabilitation group, but there were no significant differences between groups at 3, 6, 12 or 24 months, indicating that early rehabilitation might result in a faster recovery without detrimental effects in the longer term. In addition, Mazuquin et al. [18] conducted a review of systematic reviews and concluded that early mobilisation might be beneficial but suggested more high-quality studies are required, especially for patients with large tears.

## Methods/design

This protocol is reported with reference to the SPIRIT checklist (Additional file 1) [19].

### Study design

**Fig. 1:**
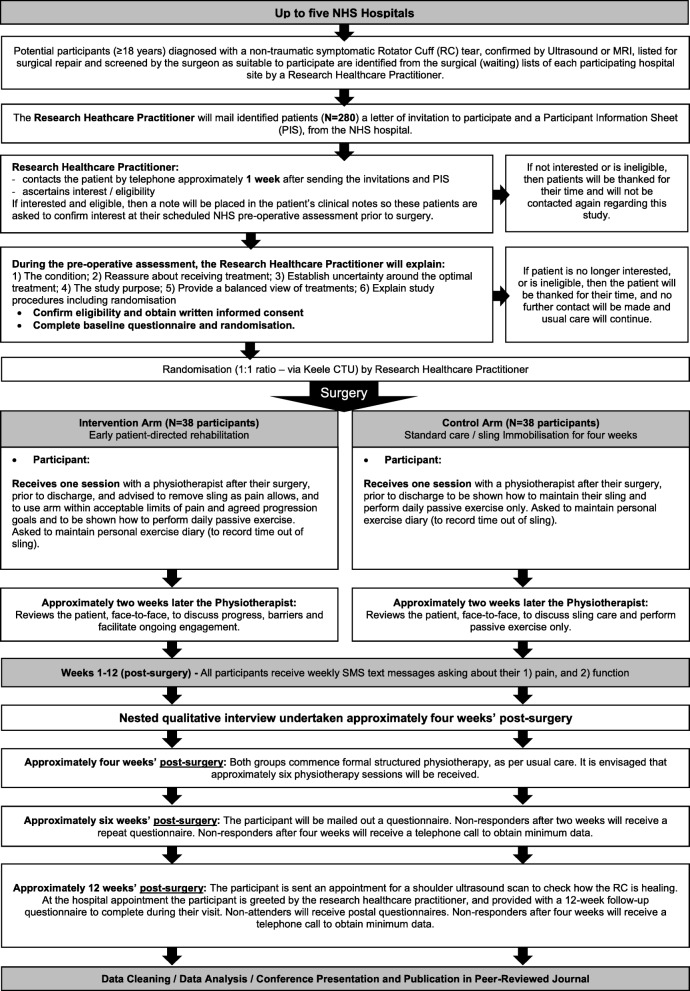
Study flow chart

**Fig. 2:**
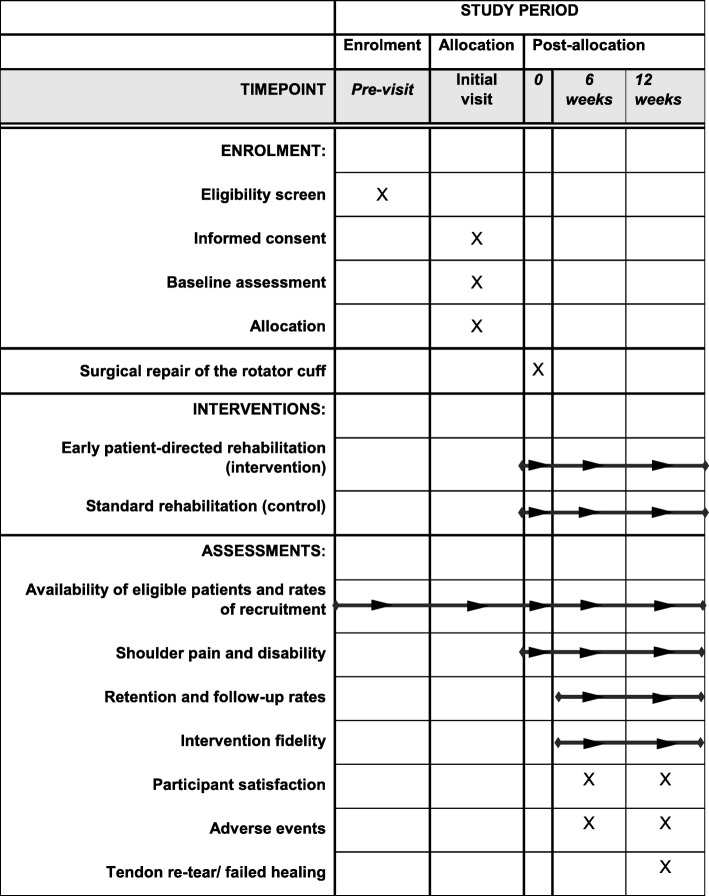
Schedule of enrolment, interventions, and assessments (SPIRIT Statement) [19]

The RaCeR study is designed as a two-arm, multi-centre pilot and feasibility RCT with nested qualitative interviews (Figs. 1 and 2).

### Aim

In patients undergoing surgical repair of the RC, we aim to assess the feasibility of a future, substantive, multi-centre RCT to test the hypothesis that, compared to delayed rehabilitation incorporating sling immobilisation, early patient-directed rehabilitation is both more clinically effective and more cost-effective.

### Objectives

The objectives of this pilot and feasibility RCT are as follows:

1. To determine the availability of eligible patients and the rate of recruitment.
2. To determine the rate of retention and the response rates to questionnaires and SMS text messages.
3. To determine whether the interventions can be delivered per protocol, including an assessment of adherence, i.e. the time out of sling for both the early rehabilitation group and the sling immobilisation group.
4. Determine the primary outcome measure for the main trial by evaluating sensitivity to change and assess other statistical properties related to clinical outcomes (e.g. standard deviation, betweenmeasurement correlations, event rate) that would be required to inform the sample size calculation for a main trial as well as an assessment of the completeness of follow-up between different methods of data capture (questionnaire and SMS text),
5. Determine rate of response and completion of healthcare resource use questionnaire and productivity, including return to work,
6. Explore participant satisfaction with interventions,
7. Determine rate of tendon re-tear or failed healing (same outcome that RC is not intact) at 12-weeks post-surgery determined via diagnostic ultrasound,
8. Determine number and nature of adverse events via self-report questionnaire.

Semi-structured qualitative interviews will be undertaken:

1. To determine the acceptability of the interventions to patients and clinicians.
2. To identify the barriers and enablers to adherence.
3. To identify which outcome measures best reflects the participants’ rehabilitation goals.

### Study setting

Patients will be recruited from orthopaedic departments of the participating UK NHS hospitals. Following surgery to repair the RC, the study interventions will be delivered prior to discharge while the patient is on the hospital ward. Subsequent components of the study interventions will be delivered through outpatient physiotherapy settings in, or affiliated to, the main hospitals.

### Participants

#### Inclusion criteria

Patients can be included if they meet all of the following criteria:

1. They have been diagnosed with a non-traumatic symptomatic tear of the RC and listed for surgical repair.
2. They have RC tear that has been confirmed by ultrasound or magnetic resonance imaging (MRI).
3. They are aged ≥18 years.
4. They have been screened by a surgeon as suitable for participation.
5. They can return to the recruiting centre or affiliated site for the initial outpatient follow-up physiotherapy appointment.
6. They have access to a mobile phone and are willing and able to receive and respond to SMS text messages
7. They understand English.

#### Exclusion criteria

Patients are excluded if they met any of the following criteria:

1. They have a traumatic RC tear (e.g. sudden onset of shoulder pain and weakness following a fall). Given the different care pathways in the UK, such patients are typically fast-tracked to surgery.
2. They unable to give full informed consent.

#### Recruitment

Potential participants will be identified from the surgical waiting list by a hospital-based research health-care practitioner (e.g. a nurse, physiotherapist or radiographer) and screened with reference to the inclusion and exclusion criteria. If potentially eligible, they will be sent in the post from the NHS hospital an invitation to participate in the study, an explanatory letter and a participant information sheet. These will include contact details of the research health-care practitioner. They will then be followed up by the research health-care practitioner within approximately 1 week by telephone (up to three attempts) or at a different time if the patient wishes to initiate the telephone call. If during screening including the telephone call, the patient indicates an interest in being involved or wishes to have further time to consider, they will be invited to discuss participation in the study further with the research health-care practitioner at the time of the pre-operative clinic appointment or another convenient time. For patients who cannot be contacted over the telephone, a follow-up letter will be sent asking them to make contact with the research health-care practitioner at the time of their pre-operative assessment should they be interested.

This discussion will build on a recent successful six-step model to promote recruitment to RCTs that resulted in 57% recruitment of eligible patients in an orthopaedic setting [20], and will include: (1) an explanation of the condition, (2) reassurance about receiving treatment, (3) telling the patient about the uncertainty in what is the most effective approach to rehabilitation to highlight reasons why the research is necessary, (4) an explanation of the purpose of the study, (5) a balanced view of the two interventions after surgery and (6) an explanation of study procedures including randomisation and follow-up requirements. This process will be supported by a recruitment script written for the research health-care practitioner, which they can refer to during both the initial telephone call and the subsequent pre-operative clinic appointment.

During the telephone call, medical note review and face-to-face meeting, the patient’s age and year of birth, gender, diagnosis, size and location of RC tear and, if applicable, reason for exclusion will be collected. Once patients have been identified, and it is confirmed that they have a symptomatic RC tear and are 18 years or older, the research health-care practitioner will check their medical notes to confirm that the surgeon responsible for their care does not wish them to be excluded from participation in the study prior to contacting them. This checking process will be documented.

Interested and eligible patients will be required to provide written informed consent before participating. Typically, written informed consent will be sought at the time of the pre-operative assessment or other convenient time prior to surgery. A research health-care practitioner, who will have received appropriate training and is authorised on the trial delegation log, will take informed consent. A record of the consent process will be kept in the patient’s medical records. The original consent form will be retained in the study site file and a copy will be given to the participant. A copy will also be securely sent to Keele Clinical Trials Unit (CTU) for monitoring purposes.

Participants have the right to withdraw at any time without any consequence to any future care they may require. For each trial participant, their general practitioner will be sent a letter from Keele CTU to confirm that their patient is taking part in the research study.

### Data collection

The research health-care practitioner will provide the baseline questionnaire to consenting patients for them to complete themselves. This collects demographic data, general practitioner details, the Oxford Shoulder Score (OSS), the Shoulder Pain and Disability Index (SPADI) and EQ-5D-5L. The patient will be given adequate time and a suitable location to complete the baseline questionnaire. It will be checked for completeness by the research health-care practitioner. The original baseline questionnaire will be sent to Keele CTU and a copy retained in the study site file. Once the baseline questionnaire has been completed and checked, the participant will be randomised to either the intervention or control group.

The size and location of the tear will be recorded, since these may be important determinants of the clinical outcome. This information will be collected by the research health-care practitioner from the pre-operative imaging, ultrasound scan (USS) or MRI, which will typically be available in the medical notes. Due to concerns about the reliability of measurements of the size and location of a tear by imaging alone, the physiotherapist who delivers the allocated treatment will record the intra-operative findings of the size and location of the tear and the completeness of repair as they will typically have access to this information. The completeness of the repair will allow the study team to contextualise the USS 12 weeks post-operatively. Previous studies have been criticised for reporting re-tears only dichotomously, i.e. torn or not, whereas a re-tear identified on an USS might just reflect that the tear was only partially repaired or not repaired at the time of the operation.

The OSS is a 12-item shoulder-specific self-report measure of shoulder pain and function, primarily used to assess the outcome of shoulder surgery in RCTs [21]. The OSS is reliable, valid, responsive and acceptable to patients [21–23]. The items refer to the past 4 weeks. Each has five ordinal response options scored from 0 to 4, with 4 representing the best outcome. When the 12 items are summed, this produces an overall score ranging from 0 to 48, with 48 being the best outcome.

The SPADI is a self-report measure of pain and function in patients with shoulder disorders [24]. It is the most commonly used outcome measure in trials of conservative interventions [25]. The SPADI has been validated for use with patients who have shoulder pain. A minimally clinically important change of 10 points has been identified [24, 26]. Additionally, excellent levels of reliability (intraclass correlation coefficient 0.66 to 0.95), high internal consistency (Cronbach’s α typically >0.9) and responsiveness over time have been reported in tandem with no floor and ceiling effects [26]. The SPADI has 13 items divided into two sub-scales: pain (5 items) and disability (8 items). The responses are indicated on a visual analogue scale where 0 = *no pain/no difficulty* and 10 = *worst imaginable pain/so difficult it requires help*. The items are summed and converted to a total score out of 100 where a high score indicates greater pain and disability.

The EQ-5D-5L is a generic measure of health-related quality of life. It provides a single index value for health status that can be used in a clinical or health economic evaluation [27]. The EQ-5D-5L consists of questions relating to five health domains (mobility, self-care, usual activities, pain/discomfort and anxiety/depression) and respondents rate their degree of impairment using five response levels (no problems, slight problems, moderate problems, severe problems and extreme problems) [27]. The EQ-5D-5L is the National Institute for Health and Care Excellence’s preferred measure of health-related quality of life in adults.

A patient-completed personal exercise diary will be returned to the treating physiotherapist at the 4-week follow-up appointment and then returned to Keele CTU. Alternatively, it can be returned to Keele CTU along with the 6-week postal questionnaire, if necessary.

The rate of tendon re-tear or failed healing will be evaluated through a diagnostic USS performed at 12 weeks according to an agreed and standardised protocol for the sonographers. This information will be compared to the pre-operative imaging results and the intra-operative findings. A diagnostic USS is regarded as a relatively cheap but accurate modality for identifying RC tears [28].

### The randomisation scheme

Blocked randomisation in a 1:1 ratio will be undertaken remotely using web-based randomisation supported by Keele CTU to ensure allocation concealment. Following confirmation of eligibility, and the receipt of a correctly completed and signed consent form and a completed baseline questionnaire, an authorised research health-care practitioner will access Keele CTU’s secure system to randomise the participant to one of the two treatment arms. The randomisation will be performed using random permuted blocks and will be blocked by recruiting site (block sizes of 2 and 4) to ensure that patients at each centre have an equal chance of receiving either treatment. The allocation will be placed in a sealed opaque gold envelope that is attached to the patient’s medical notes or added as a note to the electronic patient file, according to the local procedure. The envelope or note will be addressed to the physiotherapist who will deliver the treatment.

### Blinding

The participant will be informed of the allocation only by the physiotherapist who will deliver the allocated treatment, and the surgeon will remain unaware of the allocation until after the surgery to minimise the likelihood of withdrawal through knowledge of the allocated intervention. No further measures to blind participants, clinicians, research team or oversight committees will be implemented in this external pilot and feasibility RCT.

#### Interventions

##### Control group

Reflective of current UK practice [7], after surgery, the patient’s shoulder will be immobilised using a sling for 4 weeks. They will have one session in the hospital with a physiotherapist, after surgery but prior to discharge. This session will include advice to maintain the sling in situ at all times. It should be removed *only* to perform specific daily exercises (table slides, active elbow, wrist and hand exercises and passive shoulder abduction, flexion and lateral rotation movements, within pain limits), or for eating, washing and dressing. The sling should also be worn at night. No further active movement will be encouraged. A post-operative information and exercise booklet with photographs and text detailing the exercise type and number of sets and repetitions will be provided. This initial discussion will be followed up approximately 2 weeks later at an outpatient appointment with the physiotherapist, who will reconfirm this advice.

Adherence to the programme, i.e. time out of the sling, will be measured using a personal exercise diary. This was developed in association with our patient and public involvement and engagement (PPIE) group and will be provided by the physiotherapist. Participants will be asked to record in the diary for how long they were not wearing the sling at regular periods throughout the day, in the same way as the intervention group. Participants will also be asked to record the extent to which they feel they have adhered to the prescribed exercise plan.

##### Intervention group

The intervention group will undergo early patient-directed rehabilitation. This includes one session in hospital with a physiotherapist, after surgery but prior to discharge. In contrast to the control intervention, this session will include advice to remove the post-operative sling, as pain allows, as soon as possible and gradually to begin actively using the arm within the limits of their pain. This use will increase over time according to agreed goals within the context of the patient’s own pain experience and tolerance. Like the participants in the control group, participants in the intervention group will also receive advice to perform specific daily exercises (table slides, active elbow, wrist and hand exercises, and passive shoulder abduction and flexion movements, all within pain limits). However, given that the intention of the intervention is to encourage early movement after surgery, if participants remove their arm from the sling at times other than to exercise, then they will not need to do the basic table slides and active elbow, wrist and hand exercises. Instead, they will be advised that only shoulder abduction, flexion and lateral rotation movements are required to encourage range of movement. A post-operative information and exercise booklet with photographs and text detailing the exercise type and number of sets and repetitions will be provided.

This initial discussion will be followed up approximately 2 weeks later at an outpatient appointment with a physiotherapist to discuss progress, address barriers to sling removal and active functional shoulder movement, and to facilitate adherence with the early rehabilitation. As with the control group, an assessment of the implementation of and adherence to the intervention will be facilitated by the personal exercise diary, which the participant will use to record the time out of the sling at regular periods throughout the day. Participants will also be asked to record the extent to which they feel they have adhered to the prescribed exercise plan.

##### Both groups

Both groups will be provided with the same type of sling and advised to take pain medication as prescribed. Use of medication will be self-reported and analysed. After 4 weeks, both groups will commence 1:1 physiotherapy as per current UK practice, which includes a progressive exercise programme targeting range of movement, strength and function [7] as follows:

- Phase 1: Progress to assisted movement (shoulder movement with assistance) then full active movement (shoulder movement by the patient) within pain limits
- Phase 2: Isometric exercises (resisted static exercises) for all shoulder muscle groups
- Phase 3: Resisted exercises through range, within limits of pain
- Phase 4: Functional restoration

This physiotherapy protocol has been agreed with all clinical sites. Progress between the phases will be individualised and guided by the patients’ acceptable symptom response over four further physiotherapy sessions (six in total) complemented by a home exercise programme. Delivery of the intervention and control treatments will be supported by a manual for the physiotherapists.

#### Study training

Research health-care practitioners will be trained in each of the participating centres on the participant eligibility criteria, the approach to recruitment and consent, the requirements for the completion of all study paperwork, good clinical practice as applicable to research and the maintenance of the study site file and study records. Reporting of serious adverse events and adverse events will also be covered.

In addition, the clinical physiotherapists, both those who will initially prescribe the study intervention to patients on a ward in an in-patient setting and those who will subsequently assess and treat patients in an outpatient setting, will be trained prior to the start of recruitment and treatment. Due to the limited number of clinical physiotherapists within the clinical site teams, each clinical physiotherapist will treat patients in both arms. However, given the protocolised nature of the interventions, we do not expect a significant contamination effect and we will include contamination and how to avoid it within the study training.

The focus of this training will be on delivery of the interventions and will be supplemented by a comprehensive manual providing clear treatment protocols and guidance on study paperwork for the clinicians. They will also receive instruction sheets for the exercises, personal exercise diaries for participants and post-operative patient information booklets with information on returning to usual activities, including driving and work. The training will take the form of workshops consisting of information provision, question and answer sessions and role play with regards to supporting the approach to early patient-directed rehabilitation and overcoming any barriers.

The sonographers will undertake the USS of the shoulder at 12-weeks to assess whether the RC has re-torn. The USS will be focused on the RC and details of the RC tear and surgery will be provided, as described above. The sonographers will be provided with information regarding the RaCeR study and will be offered the opportunity to discuss the protocol with the research team. However, because the protocol reflects usual practice in the UK, further formal training will not be routinely offered.

### Outcomes

#### Feasibility outcomes

1. Numbers of potentially eligible and eligible patients, number invited, number attending a pre-operative assessment clinic and consent rates (target recruitment is six or seven participants per month over a 12-month recruitment period)
2. Number of eligible patients found subsequently during surgery not to have an RC tear (indicating a false positive scan)
3. Feasibility of recruiting participating centres and number of additional centres in which staff are interested in participating in a future main trial
4. Rate of retention in the trial, including response rates to questionnaires and individual measures (including resource use) and SMS text messages
5. Intervention fidelity and adherence using physiotherapist-completed case report forms and patient-completed personal exercise diaries for time out of sling
6. Determining the primary outcome measure for the main trial by assessing and comparing sensitivity to change of the measures and other statistical properties to support sample size derivation, comparing follow-up rates of different methods of data capture (questionnaire/SMS text) and of individual measures, and evaluating which outcome measures best reflect the patients' rehabilitation goals, identified from the qualitative interviews,
7. Participant satisfaction with the interventions on a five-point ordinal scale, ranging from very satisfied, satisfied, neutral and dissatisfied to very dissatisfied
8. Patient and clinician views about the acceptability of the interventions (qualitative data)

#### Clinical outcomes

1. Pain and disability assessed using the OSS and SPADI at baseline, 6 weeks post-surgery by post and 12 weeks post-surgery in person during a follow-up clinic visit or by postal questionnaire, or by telephone call to collect minimal data if there is no response to the postal questionnaire
2. Health-related quality of life assessed using the EQ-5D-5L at baseline, 6 weeks post-surgery by post and 12 weeks post-surgery in person during a follow-up clinic visit or by postal questionnaire
3. Global change question at 6 weeks post-surgery by post and 12 weeks post-surgery in person during a follow-up clinic visit or by postal questionnaire, or by telephone call to collect minimal data if there is no response to the postal questionnaire
4. Pain in the last week (derived from the OSS) using weekly SMS text messages for 12 weeks post-surgery based on a 0–4 numerical rating scale with anchors of *no pain* (0) to *unbearable pain* (4)
5. Disability relating to work or activity interference due to the shoulder problem in the last week (derived from the OSS) using weekly SMS text messages for 12 weeks post-surgery based on a 0–4 numerical rating scale with anchors of *not at all* (0) to *totally* (4)
6. Days lost from work due to the shoulder problem at 6 weeks post-surgery via postal questionnaire and 12 weeks post-surgery via questionnaire completed during a follow-up clinic visit
7. Time taken to return to driving, if applicable, via a questionnaire at the 6- and 12-week follow-ups
8. Number and type of adverse events, e.g. post-procedural exacerbation of pain, for up to 12 weeks post-randomisation via patient self-report questionnaire at 6 and 12 weeks post-surgery and via surgeon, physiotherapist or general practitioner report
9. Diagnostic USS performed by a sonographer independent of the study to assess the integrity of surgical repair at 12 weeks post-surgery

We will also collect self-report data relating to further health-care resource use, including NHS- and private-borne service and medication costs as well as data from the surgery itself, including the completeness of the surgical repair, and questions relating to the patient’s occupation and their loss or gain of productivity at work.

### Sample size calculation

Although RaCeR is a pilot and feasibility RCT, a sample size calculation has been conducted to ensure there is a sufficient number of participants to detect a difference in time out of sling between the two randomised groups. Understanding this is a key feasibility objective and will be an important factor in determining whether a future main trial is feasible. Randomising 76 participants, which allows for 20% missing adherence-related data (time out of sling captured using the personal exercise diaries), will provide 90% power to allow us to detect at least a minimum 40% difference in sling use between the two arms, given a 1-sided 5% significance level. Randomising 76 participants will enable us to estimate the 1-sided lower 90% confidence limit for the follow-up rate to within about 6% of the anticipated 80% level. Further, a sample size of about 76, allowing for dropouts, will be sufficient to allow precise calculation of estimates of the standard deviations around potential primary clinical outcomes for a future main trial [29].

#### Planned recruitment rate

The planned recruitment rate is six or seven participants per month over a 12-month period to reach the target of 76.

### Statistical analysis plan

As this is a pilot and feasibility RCT, the main analysis will focus on process outcomes and will estimate the number of potentially eligible patients, the number and rate of eligible patients, the consent rate, the overall uptake rate (in relation to the number potentially eligible), the retention rate, follow-up rates for questionnaires and texts, and adherence to the interventions, including time out of sling. We will calculate means and confidence intervals of clinical outcomes (both within- and between-study groups) and determine which outcomes are most sensitive to change. We will evaluate the parameters required to inform the sample size calculation for a future main trial. A detailed statistical analysis plan will be developed by the study statistician and agreed by the trial steering committee.

At the end of the study, we will review the findings and in discussion with the trial steering committee, we will make a recommendation about proceeding to a future main trial. The following success criteria will be used to decide whether to make an application for funding for a main trial:

i.) Recruitment rates: Randomisation after consent of 20% or more of eligible patients
ii.) Intervention adherence: Difference in time out of sling of 40% or more, i.e. the early rehabilitation (intervention) group will report 40% more time out of the sling than the control group in the first 4 weeks after surgery
iii.) Follow-up rates for the potential main outcome measures >70% for questionnaires at 6 and 12 weeks and weekly for the SMS text messages

### Patient and public involvement and engagement

We have held three structured PPIE group meetings facilitated by the lead researcher (CL). One group comprised patients who had undergone surgery to repair their RC and the other two meetings were with experienced PPIE members drawn from the Research User Group at Keele University. Additionally, one PPIE member (TC) is a co-applicant and co-author and continues to contribute to this project regularly.

The aims of PPIE were multiple, including developing study processes (for example, recruitment), developing patient-facing materials (for example, the participant information sheets) and contributing to study management and oversight (for example, membership of the trial management group and trial steering committee).

PPIE resulted in development of the process of recruitment whereby it was suggested that recruitment and the process of informed consent be moved away from the clinical consultation to a separate time facilitated by a research healthcare practitioner. Study materials, for example the personal exercise diary, were also developed to be more user-friendly.

PPIE has resulted in tangible outcomes that will facilitate study delivery, including recruitment and data collection. In addition, PPIE has an ongoing and important contribution to trial management and oversight [30].

### Qualitative interviews

We anticipate interviewing up to 10 patient participants from each of the two groups (up to *n* = 20 in total) and up to 10 clinician participants (shoulder surgeons (approximately *n* = 3), physiotherapists (*n* = 4) and nurses (approximately *n* = 3)) involved in explaining the trial to patients, conducting the screening and consent processes, or delivering the intervention. We expect that this number of patients and clinicians will be sufficient to reach data saturation. We will cover similar topics with all interviewees, to ensure that we can compare their responses, while at the same time enabling them to reflect on their specific expectations, understanding and experiences.

Information about the qualitative interviews will be included in the initial RCT participant information sheet. The RCT consent form will include consent for the patient to be contacted post-surgery to discuss their participation. Patients will be purposefully sampled post-surgery with respect to their allocated treatment group and their pain and disability status according to early SMS text message responses. They will then be contacted by telephone to discuss their further participation in the qualitative study. If a patient wishes to participate, then a participant information sheet pertaining specifically to the qualitative study and a consent form will be posted to them with a pre-paid envelope. On receipt of their written informed consent, a mutually convenient time to conduct the interview will be arranged. Patients may decline to participate in the interviews yet still be involved in the trial.

The clinicians will be informed of the interviews during the initial site set-up and follow-up visits. Clinician participant information sheets and consent forms will be provided, allowing them to consent to be contacted. If a clinician consents to be contacted, the research team will subsequently contact them with a view to discussing participation once they have gained experience in explaining the trial to patients, in the screening and consent processes, or in delivering the intervention. If a clinician wishes to participate then, on receipt of their written informed consent, a mutually convenient time to conduct the interview will be arranged.

Patient participants will be interviewed around 4 weeks after their surgery. The interviews will last for approximately 45 minutes and be face-to-face or by telephone. All interviews will be conducted by an experienced qualitative researcher and will be based on semi-structured topic guides developed in association with our PPIE group. A similar process will be followed for the clinician participants.

With the participants’ consent, interviews will be audio-recorded and transcribed verbatim. In the unlikely event that an audio-recording is of poor quality, the participant will be re-contacted and invited to a second interview. The interviews will be sent securely to and transcribed by a professional transcription company, which operates under the terms of a confidentiality agreement. Transcripts will be anonymised during the transcription process.

Interviews will be analysed both thematically (indexing key themes and contextualising these in the broader data set) and as narratives (capturing the patient journey for each patient participant). This approach ensures that the context of individual patient journeys is preserved while supporting a broader thematic analysis [31]. The software NVivo will be used. We will develop an appropriate coding strategy and coding framework that facilitates data retrieval and comparative analyses [32].

## Discussion

The evidence to date suggests there is the potential to reduce the patient burden associated with post-operative immobilisation following surgery to repair a torn RC and to improve clinical outcomes. The principal concern about early rehabilitation is the perceived risk of re-tearing the repaired RC, but this concern might be unfounded. Given an ageing population, the increasing prevalence of RC disorders, the rising rates of RC surgery and preliminary evidence that early rehabilitation might improve clinical outcomes, further research is now needed. There is a clear need for a high-quality, adequately powered, randomised trial evaluating outcomes including pain, disability and the re-tear rate to inform clinical practice. Prior to the main trial, this RaCeR pilot and feasibility trial will first address the current uncertainties about recruitment, retention and adherence with the intervention, particularly in relation to whether patients will be willing to begin moving their arm early after their operation.

### Trial status

At the time of submission of this protocol paper, RaCeR (protocol version 2.0 dated 26 July 2018) was open to recruitment (commenced recruitment 21 November 2018). Recruitment is scheduled to be complete by 20 November 2019.

## Additional file

Additional file 1: SPIRIT 2013 Checklist: Recommended items to address in a clinical trial protocol and related documents. (DOC 119 kb)
